# The endocannabinoid anandamide prevents TH17 programming of activated T lymphocytes while preserving TH1 responses

**DOI:** 10.3389/fphar.2024.1528759

**Published:** 2024-12-20

**Authors:** Anastasiia Kiprina, Tom Teichmann, Virna Margarita Martín Giménez, Wenqing Xu, Fiona Sailer, Maike Windbergs, Walter Manucha, Andreas Weigert, Ralf P. Brandes

**Affiliations:** ^1^ Institute of Biochemistry I, Faculty of Medicine, Goethe University Frankfurt, Frankfurt, Germany; ^2^ Institute for Cardiovascular Physiology, Goethe University Frankfurt, Frankfurt, Germany; ^3^ German Centre of Cardiovascular Research (DZHK), Partner site RheinMain, Frankfurt, Germany; ^4^ Instituto de Investigaciones en Ciencias Químicas, Facultad de Ciencias Químicas y Tecnológicas, Universidad Católica de Cuyo, San Juan, Argentina; ^5^ Institute of Pharmaceutical Technology, Goethe University Frankfurt, Frankfurt, Germany; ^6^ Instituto de Medicina y Biología Experimental de Cuyo (IMBECU), Consejo Nacional de Investigaciones Científicas y Tecnológicas (CONICET), Mendoza, Argentina; ^7^ Departamento de Patología, Área de Farmacología, Facultad de Ciencias Médicas, Universidad Nacional de Cuyo, Mendoza, Argentina

**Keywords:** AEA, endocannabinoids, T cells, inflammation, lipids

## Abstract

**Introduction:**

Anandamide (AEA) is an endocannabinoid that has recently been recognized as a regulator of various inflammatory diseases as well as cancer. While AEA was thought to predominantly engage cannabinoid (CB) receptors, recent findings suggest that, given its protective anti-inflammatory role in pathological conditions, anandamide may engage not only CB receptors.

**Methods:**

In this study, we studied the role of exogenous AEA in a mouse AirPouch model of acute inflammation by examining immune cell infiltrates by flow cytometry. Human primary immune cells were used to validate findings towards immune cell activation and migration by flow cytometry and bead-based ELISA.

**Results:**

We found that AEA decreases the acute infiltration of myeloid cells including granulocytes and monocytes into the inflamed area, but unexpectedly increases the number of T cells at the site of inflammation. This was related to AEA signaling through nuclear receptor subfamily 4A (NR4A) transcription factors rather than CB receptors. Exploring regulatory mechanisms in the human system, we found that AEA broadly inhibits the migratory capacity of immune cells, arguing for blocked emigration of T cells from the inflamed tissue. Taking a closer look at the impact of AEA on T cells revealed that AEA profoundly alters the activation and exhaustion status of CD4^+^ T and CD8^+^ T cells, thereby strongly inhibiting TH17 responses, while not altering TH1 differentiation.

**Discussion:**

These data suggest that AEA has the potential to block chronic inflammation without influencing crucial anti-viral and anti-microbial immune defense mechanisms, and may therefore be an attractive molecule to interfere with the establishment of chronic inflammation.

## Introduction

Endocannabinoids are ligands of the G-protein-coupled endocannabinoid receptors CB1 and CB2 (Lu and Mackie, 2021). The most studied endocannabinoid is N-arachidonoylethanolamine (anandamide, AEA), which is synthesized *de novo* by cells in response to activation (Lu and Mackie, 2016). AEA has a low half-life in tissue as it is rapidly degraded by fatty acid amide hydrolase (FAAH), cyclooxygenase 2 (COX2), lipoxygenases (LOX) and cytochrome P450 (CYP) enzymes (Maccarrone, 2017).

CB receptors are also expressed outside the brain and elicit a broad spectrum of effects, among them modulation of inflammatory activity. In fact, genetic deletion of CB1 in mice has been shown to promote chronic heart failure (Liao et al., 2013), whereas CB2 results in an increased risk of atherosclerosis (Netherland et al., 2010) as well as cardiomyopathy (Duerr et al., 2014). Deletion of CB1 receptors in myeloid cells limits atherosclerosis development in male mice (Wang et al., 2024) and CB1 receptor activation promotes vascular smooth muscle cell proliferation and neo-intima formation (Molica et al., 2013). Although CB receptors are considered the predominant mediators of AEA signaling, it became clear that they are not the only signaling receptors responding to this nitro-lipid. In fact, extracellular AEA can also bind and activate the transient receptor potential vanilloid type-1 (TRPV1) and transient receptor potential ankyrin type-1 (TRPA1) channels as well as the G-protein coupled receptors GPR55 and GPR119 (Ligresti et al., 2016; Maccarrone, 2017; Pertwee et al., 2010; Zygmunt et al., 1999).

AEA is produced within the central nervous system (CNS), where it was shown to have anti-inflammatory and neuroprotective effects in the context of neuroinflammation (Eljaschewitsch et al., 2006). Moreover, AEA is known to reduce vascular, skin and endotoxin-induced inflammation (Martín Giménez et al., 2022; McCormick et al., 2023; Tomczyk et al., 2021) by acting on CB receptors, but also by triggering epigenetic changes (Martín Giménez et al., 2022). Various studies have demonstrated that AEA has oncoprotective activity against breast, prostate and non-melanoma skin cancers (Sarfaraz et al., 2008; Soliman and van Dross, 2016).

We recently identified that AEA elicits a strong anti-inflammatory effect in vascular smooth muscle cells (VSMCs), which was mediated by an epigenetic modulation through NCoR1 (Pflüger-Müller et al., 2020). Interestingly, we also observed that these effects of AEA require high concentrations and were not mediated by classic AEA receptors, like CB1 and CB2. This constellation may suggest an action of AEA through nuclear receptors (NR). NRs are proteins with transcription factor properties that are typically activated by lipophilic compounds (Frigo et al., 2021). After ligand binding and, if required, nuclear translocation, nuclear receptors, acting as mono or hetero- and homo-dimer, activate gene expression (Martínez-González et al., 2021). In addition to these classic hormone receptors, a broad spectrum of lipid receptors like peroxisome proliferator-activated receptor (PPARs) alter gene expression in response to some poly unsaturated fatty acids (PUFAs) and other ligands (O'Sullivan, 2007). Finally, orphan NRs exist and and some of them even lack a ligand binding site rendering their activity controlled through phosphorylation or abundance and subcellular localization (Mullican et al., 2013). Receptors of the NR4 class belong to the latter group, although more and more compounds are being identified, which appear to bind these receptors and increase their activity. Among them are signaling lipids like prostaglandin A2 or pharmacological compounds like CDIM12 or cytosporone B (Hammond et al., 2015; Rajan et al., 2020; Zhan et al., 2008). We recently reported that AEA binds and activates NR4A1 and NR4A2 to mediate an anti-inflammatory effect in vascular smooth muscle cells (VSMCs) (Teichmann et al., 2024). In fact, there is a good amount of data linking NR4 receptors to inflammatory control: In inflamed human synovial tissue, multiple sclerosis or atherosclerotic lesions, their expression is drastically increased (McMorrow and Murphy, 2011), while in mice, both loss of NR4A1 or NR4A2 was associated with increased inflammation (Bonta et al., 2006; Hamers et al., 2013). Moreover, NR4A receptors are rapidly and strongly induced by various inflammatory cytokines, suggesting a protective role in an acute scenario by helping to resolve inflammation through a negative feedback mechanism, aiming to restore homeostasis in the later stages of inflammation (Rodríguez-Calvo et al., 2017).

A limitation of our previous study on the AEA-mediated activation of NR4 receptors was, that the physiological relevance of the anti-inflammatory effect was only determined in organ culture of the isolated mouse aorta and cultured VSMC. In the present study we therefore set out to determine whether AEA also limits inflammation *in vivo*. Unexpectedly, we observed a strong, NR4-dependent effect of AEA on T-lymphocytes, which, among others, resulted in a prevention of differentiation towards a TH17 phenotype and rather maintained competence of the cells to respond to acute inflammatory stimulation.

## Materials and methods

### Animals

Global knockout mice for NR4A1^−/−^(Nur77), NR4A2^−/−^ (Nur1) and double knockout for NR4A1/2^−/−^ were generated by crossing NR4A1^flox/flox^ (obtained from the Jackson Laboratory) or NR4A2^flox/flox^ mice (kindly provided by Pierre Chambon (Sekiya et al., 2011), with CMV-GT-Rosa-CreERT2^TG/0^. All knockout animals were generated on the C57BL/6 background. Global deletion of NR4A1 (A1KO) and/or NR4A2 (A2KO) was induced by administering tamoxifen (400 mg/kg in chow) for 10 days, followed by a 14-day tamoxifen-free “wash-out” period. In this study, control animals (WT) are defined as littermates, which did not receive tamoxifen treatment with the chow. All animals had free access to chow and water in a specified pathogen-free facility with a 12 h light/dark cycle and all animal experiments were performed in accordance with the German animal protection law and were carried out after approval by the local authorities (Regierungspräsidium Darmstadt, approval number FU1268). Every mouse received an identification number for each experiment and the experimenter was blinded for the genotype. Animal group sizes differed due to number of available littermates.

### Preparation of AEA micellar nanoformulations

AEA is an oily substance, which limits application *in vivo* and controlled absorption. Therefore, the compound was applied as nanoformulation. The preparation of the AEA micellar nanoformulations was carried out as previously described (Martín Giménez et al., 2023). Briefly, 30 mg of the commercial co-polymer Pluronic^®^ F127 (PF127; BASF, CABA, Buenos Aires, Argentina) was accurately weighed and dissolved in 1 mL of Milli-Q water (Sigma-Aldrich, St. Louis, MO, USA) each. The mixture was continuously stirred at RT until homogeneous and a transparent dispersion was obtained. Subsequently, 750 µg of AEA, dissolved in absolute ethanol (using 15 µL of a commercial AEA ethanolic solution from Cayman Chemical, Ann Arbor, MI, USA), was incorporated (drop by drop) into the polymeric dispersion. Stirring was maintained until complete ethanol evaporation. AEA-free micellar nanoformulations of the PF127 polymer (Pluronic) served as control (CTL).

### AirPouch model of acute inflammation

The AirPouch model (Paul-Clark et al., 2012; Pierron et al., 2023) was conducted on both tamoxifen-treated mice following intake and washout, as well as on untreated mice (CTL) that did not receive tamoxifen. Mice were anesthetized using isoflurane inhalation and placed on a heating pad to maintain body temperature. The dorsal skin region was shaved, and the skin was sterilized with 70% ethanol. An AirPouch was created by subcutaneous injection of 5 mL sterile air using a 23-gauge needle, followed by additional injects of 3 mL after 3 days to maintain the pouch. On day 6, 30 µg diclofenac and 10 µg AEA-Pluronic nanoparticles or Pluronic control nanoparticles without AEA (in 0.5 mL 0.9% NaCl) were injected into the pouch. Diclofenac was administered to avoid degradation of AEA by cyclooxygenases. After 1 h of pre-incubation, 1 mL of a 1% zymosan solution was injected into the pouch. After 6 h the pouch content was recovered by injection of 1 mL PBS into the pouch, massage and aspiration of the contained liquids. The concentration used for the final experiments was the result of a dose-escalation study on individual animals to determine an effective concentration.

### Primary human macrophage generation, activation, and treatment

Human peripheral blood mononuclear cells were isolated from commercially available buffy coats from anonymous donors (DRK-Blutspendedienst Baden-Württemberg-Hessen, Institut für Transfusionsmedizin und Immunhämatologie, Frankfurt, Germany) using Ficoll density centrifugation. Peripheral blood mononuclear cells were washed twice with PBS containing 2 mM EDTA and thereafter incubated for 2 h under growth conditions in RPMI 1640 media supplemented with penicillin (100 U/mL) and streptomycin (100 μg/mL) to enable adherence to culture dishes (Sarstedt, Nümbrecht, Germany). Non-adherent cells were removed. Monocytes were differentiated into naïve macrophages with RPMI 1640 media (Gibco) containing 3% AB-positive human serum (DRK-Blutspendedienst Baden-Württemberg-Hessen, Frankfurt, Germany) for at least 7 days. Differentiated macrophages were exposed to media with 1% FCS overnight. The next day, cells were incubated in RPMI 1640 media with 1% FCS alone, with EtOH as solvent for 2 h, with 10 µM diclofenac (Sigma Aldrich) for 1 h, and treated with 10 µM AEA or EtOH as solvent for 2 h. Diclofenac was used to arrest the cyclooxygenase-mediated breakdown of AEA. After treatment, macrophages were incubated with 10 μg/mL Zymosan (Sigma Aldrich) for 6 h.

### T cell isolation, activation and treatment

Primary human peripheral blood cells were isolated from buffy coats of anonymous donors (DRK-Blutspendedienst Baden-Württemberg-Hessen, Institut für Transfusionsmedizin und Immunhämatologie, Frankfurt am Main). T cells were isolated using the EasySep™ Human T Cell Isolation Kit (Stemcell Technologies) through negative selection. The purity of T cells was greater than 95%, as confirmed by flow cytometry. The cells were cultured at concentration 1 × 10^6^ cells/mL in T-cell medium (RPMI 1640, penicillin (100 U/mL), streptomycin (100 μg/mL), FCS (10%), non-essential and essential amino acids (1%), sodium pyruvate (1%) and 1% 4-(2-hydroxyethyl)-1 piperazineethanesulfonic acid (HEPES)). Cells were supplemented with human recombinant IL-2 (10 ng/mL; PrepoTech) at days 0, 2, and 4 and β-mercaptoethanol (50 μM; Gibco). Cells were cultured for up to 6 days. T cells were treated with Diclofenac for 1 h, Diclofenac and ethanol for 2 h, and Diclofenac and AEA for 2 h. After treatment, T cells were left unstimulated and stimulated with an anti-CD3/CD28/CD2 T cell activator (Stemcell) for up to 6 days. At the endpoint, supernatants were collected for cytokine measurement, and cells were analyzed by flow cytometry.

### PBMC migration assay

The migration assay was performed using 6.5 mm diameter transwell cell culture inserts (5 µm pore size; Costar). Human PBMCs were isolated from the buffy coats by Ficoll density centrifugation, washed, counted, and incubated overnight in RPMI 1640 media with 10% FCS, human recombinant IL-2 (10 ng/mL), and β-mercaptoethanol (50 μM). The next day, 10^5^ cells were seeded in the insert in serum-free medium. Prior to the migration assay, PBMCs were treated with AEA and Diclofenac or left untreated. Treated cells were used to migrate toward the medium with FCS. PBMCs that did not receive treatment were used for migration toward macrophage-derived supernatants, medium with AEA (EtOH as control), Diclofenac, and Zymosan (10 μg/mL). Cells were allowed to migrate for 3 h in cell culture. Afterwards, migrated and non-migrated cells were analyzed by flow cytometry. The percentage of migrated immune cells was determined by the ratio of migrated/non-migrated cells.

### LegendPlex

The concentrations of CCL2, CCL20, CCL4, CCL17, CCL5, IL-8, CXCL1, CXCL10, and CXCL9 in macrophage supernatants were quantified using LegendPlex (BioLegend). Samples were analyzed via flow cytometry. Data were analyzed using FlowJo V.10 (Tree Star).

### Cytometric bead array

IFN-γ, IL-10, IL-13, and IL-17A concentrations in the T cell-derived supernatants were quantified using Cytometric Bead Array flex sets (BD Bioscience). Samples were analyzed via flow cytometry. Data were analyzed using FlowJo V.10 (Tree Star).

### Flow cytometry

Single-cell suspensions from air pouches were blocked with FcR blocking reagent (Miltenyi Biotec) in 0.5% PBS-BSA for 10 min, stained with fluorochrome-conjugated antibodies (Table 1) and analyzed on a FACSSymphony A5 flow cytometer (BD Biosciences). Live single cells were identified by FSC/SSC characteristics. Data were analyzed using FlowJo V10 (TreeStar).

**TABLE 1:** Antibodies used for FACS analysis of AirPouch samples.

Antigen	Fluorochrome	Clone	Dilution used	Manufacturer	Cat. No.
CD3	PE-CF594	145-2C11	1:100	BD Biosciences	562286
CD4	V500	RM4-5	1:75	BD Biosciences	560782
CD8	BV650	53–6.7	1:100	Biolegend	100742
CD11b	BV605	M170	1:200	Biolegend	101257
CD11c	AlexaFluor700	N418	1:200	Biolegend	117320
CD19	APC/Fire 750	6D5	1:100	Biolegend	115558
CD45	VioBlue	30F11	1:50	Miltenyi Biotec	130-118-953
CD206	FITC	C068C2	1:100	Biolegend	141704
F4/80	PE-Cy7	BM8	1:200	Biolegend	123114
HLA-DR (MHC II)	APC	M5/114.15.2	1:100	Biolegend	107614
Ly-6C	PerCP-Cy5.5	HK1.4	1:200	Biolegend	128012
Ly-6G	APC-CTy7	1A8	1:100	Biolegend	127624
CD80	PE	16-10A1	1:50	Biolegend	104708

T cells and PBMCs were harvested, pelleted by centrifugation, blocked with an FcR blocking reagent (Miltenyi Biotec) in 0.5% PBS-BSA, stained with fluorochrome-conjugated antibodies (Tables 1, 2), and analyzed on a FACSymphony A5 flow cytometer (BD Biosciences). Data were analyzed using FlowJo V.10 (TreeStar).

**TABLE 2:** Antibodies used for FACS analysis of migration and T-cell activation assays.

Antibody	Clone	Provider
CD192-PE-Cy7	K036C2	BioLegend
CD184-BV750	12G5	BD Biosciences
CD183-APC	G025H7	BioLegend
CD279-BUV737	EH12.1	BD Biosciences
CD223-AlexaFlour488	11C3C65	BioLegend
CD69-BUV395	FN50	BD Biosciences
CD4-BB630	SK3	BD Biosciences
CD8-BV650	RPA-T8	BD Biosciences
CD196-PE	G034E3	BioLegend
CD38-BUV615	HIT2	BD Biosciences
TIGIT-PE-CF594	A15153G	BioLegend
CD194-BV421	L291H4	BioLegend
CD25-BUV661	2A3	BD Biosciences
CD3-BUV805	SK7	BD Biosciences
CD20-APC-H7	2H7	BD Biosciences
HLA-DR-APC Fire750	L243	BioLegend
CD56-BV605	HCD56	BioLegend
CD16-BV650	3G8	BD Biosciences
CD195-BUV395	3A9	BD Biosciences
CD198- PE-CF594	433H	BD Biosciences
CD14-BB700	MΦP9	BD Biosciences
CD33-BV510	WM53	BD Biosciences

All antibodies and secondary reagents were titrated to determine optimal concentrations. Comp-Beads (BD) were used for single-color compensation to create multicolor compensation matrices. For gating, fluorescence minus one controls were used. The instrument calibration was controlled daily using Cytometer Setup and Tracking beads (BD Biosciences). To determine the actual number of cells, counting beads were used (Bangs Laboratories).

### Statistical analysis

All experiments were independently performed at least three times as indicated by the number (n) in the respective figure legend. Statistical analysis was performed using Prism 10.1.2. Shapiro-Wilk tests were used to test for normal Gaussian distribution. A paired two-tailed Student’s t-test was used to calculate statistically significant differences between two groups of human immune cells. A one-sample Student’s t-test was used to calculate statistically significant differences between normalized data. ANOVA followed by Tukey’s test or Student’s t-test was used to evaluate statistical significance in murine data. Values of *p* ≤ 0.05 were considered significant. All data are expressed as mean ± standard error of mean (SEM).

## Results

### Anandamide increases T-cell but reduced granulocyte infiltration in the airpouch model

**FIGURE 1:**
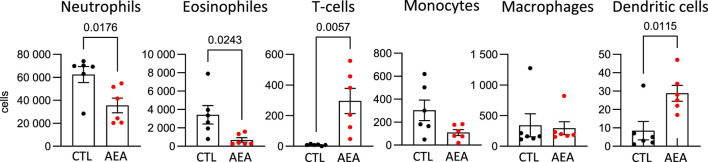
AEA increases T-cell number in AirPouch exudates in response to zymosan. Analysis of cellular infiltrate in murine AirPouch exudates after control (CTL) or AEA nanoformulation (AEA) pre-treatment (1 h) followed by zymosan stimulation (6 h) in mice. Each data point represents an animal showing the total number of cells recovered by FACS analysis. Mann-Whitney test. n = 4–8. *p < 0.05.

To determine a potential anti-inflammatory effect of AEA *in vivo* the AirPouch model of acute inflammation was used. AEA nanoformulations or nanoformulation control were injected into the pouch followed by Zymosan stimulation. Subsequently accumulation of inflammatory cells in the pouch was determined. As compared to control, the number of neutrophils and eosinophils was significantly lower in the AEA group. A similar trend existed for monocytes, but did not reach statistical significance. In contrast to this, the number of T cells as well as dendritic cells (DCs) recovered from the pouch was significantly higher in the AEA group as compared to the control group (Figure 1). Thus, AEA treatment reduced myeloid cell infiltration in the pouches, while the number of invaded T cells, which are typically involved in the later immune response, was increased by AEA.

### Anandamide directly decreases chemokine levels in Zymosan treated human macrophages

**FIGURE 2:**
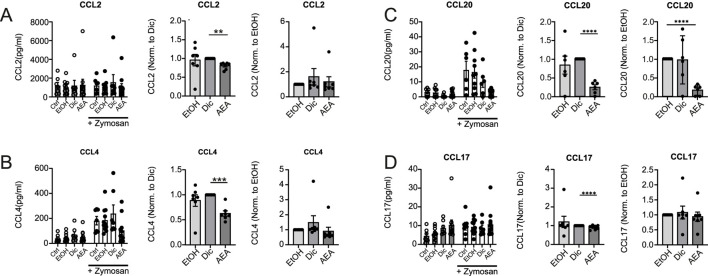
AEA reduces chemokine production in Zymosan-activated Macrophages. Human primary macrophages were treated with ethanol (EtOH), Diclofenac and EtOH (Dic), or and AEA (10 µM) with Diclofenac (AEA), and were subsequently stimulated with Zymosan for 6 h. The concentrations of CCL2 **(A)**, CCL4 **(B)**, CCL20 **(C)**, and CCL17 **(D)** were determined by LegendPlex assay. Besides raw data, normalized data of Zymosan-activated cells is shown. Data are from three independent experiments, with at least 2 donors each. Each data point corresponds to a single donor (n = 7). Data are shown as mean ± SEM. *p < 0.05, **p < 0.01, ***p < 0.001, ****p < 0.0001; *p*-values were calculated using two-way ANOVA or one-sample *t*-test.

To determine potential mechanisms explaining the findings in the AirPouch model, human primary immune cells were used. First, the impact of AEA on chemokine production by Zymosan-activated primary human macrophages was tested, since local macrophages are the first responders to Zymosan and affect recruitment of further immune cells, among other mechanisms by producing chemokines. Human macrophages were exposed to a medium containing 1% FCS overnight, followed by AEA treatment and activation with Zymosan. Initially, AEA was used at a concentration of 10 nM or 10 µM. However, 10 nM AEA did not have any impact on chemokine levels. When looking at overall chemokine levels, AEA at 10 µM did not significantly change the chemokine production in Zymosan-activated human macrophages (Figure 2; Supplementary Figure S1) due to high variability in chemokine levels between donors. However, when data were normalized, a decreased expression of CCL2 (Figure 2A), CCL4 (Figure 2B), and CCL20 (Figure 2C) in the presence of AEA compared to the Diclofenac and solvent controls (CCL2, CCL4, and CCL20), or only compared to solvent control (CCL20), was noted. Additionally, AEA decreased the production of CCL17 (Figure 2D) compared to Diclofenac and the solvent, even though the changes were minor. The expression of the remaining chemokines was consistent among control and treated groups (Supplementary Figure S1). These data suggest that high concentrations of AEA reduce the expression of chemokines, which participate in attracting cells to the site of inflammation. These findings, while being in line with reduced myeloid cell numbers, did not explain increased T cell numbers in the AirPouch model after AEA treatment. To investigate, whether changes in chemokine production directly alter the migratory capacity of immune cells, a boyden chamber assay was performed in which human PBMCs were allowed to migrate towards a supernatant derived from Zymosan-activated macrophages macrophages that were treated with either solvent controls or AEA. However, leukocyte migration towards supernatants derived from macrophages was not affect, whether they were treated with AEA or left untreated (data not shown). Unexpectedly, also CCR6+ CD4^+^ T cells and CCR6+ CD8^+^ T cells, as responders to CCL20, did not show altered migration towards supernatants from the AEA-treated as compared to control-treated macrophages. In addition, conditioned media of AEA-treated macrophages as compared to control conditions did not alter the migration capability of CCR2+ cells (data not shown). Taken together, AEA reduced the levels of the chemokines CCL20, CCL4, CCL2, and CCL17 in Zymosan-activated macrophages, but this did not directly impact immune cell migration.

### The presence of AEA suppresses the migration of human immune cells

**FIGURE 3:**
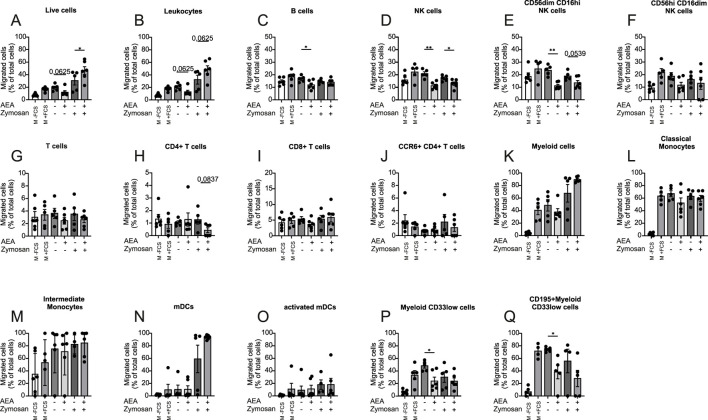
AEA and Zymosan together alter immune cell migration. Human PBMCs were kept for 3 h to migrate toward media without FCS (-FCS), with FCS (+FCS), AEA (10 µM), Zymosan, or AEA (10 µM) and Zymosan. All groups except positive and negative controls contained Diclofenac (10 µM). The numbers of migrated cells were measured by flow cytometry. The percentage of migrated live cells **(A)**, leukocytes **(B)**, B cells **(C)**, NK cells **(D)**, CD56dim CD16hi NK cells **(E)**, CD56hi CD16dim NK cells **(F)**, T cells **(G)**, CD4^+^ T cells **(H)**, CD8^+^ T cells **(I)**, CCR6+ CD4^+^ T cells **(J)**, myeloid cells **(K)**, classical Monocytes **(L)**, intermediate Monocytes **(M)**, mDCs **(N)**, activated mDCs **(O)**, myeloid CD33low cells **(P)**, and CD195^+^ myeloid CD33low cells **(Q)** was calculated by the ratio of migrated/non-migrated cells. Data are from three independent experiments, with 2 donors each. Each data point corresponds to a single donor (n = 6). Data are shown as mean ± SEM. *p < 0.05, **p < 0.01, ***p < 0.001, ****p < 0.0001; *p*-values were calculated using one-way ANOVA with Tukey correction, paired *t*-test and Wilcoxon signed-rank test for non-parametric data.

To explore a direct effect of AEA on human PBMC migration, the Boyden chamber migration assay was employed to allow PBMCs to migrate towards media containing solvent (CTL), AEA, Zymosan, and AEA + Zymosan. Diclofenac was present in each group to prevent cyclooxygenase-mediated breakdown of AEA. Media with and without FCS were used as positive and negative controls respectively, and the proportion between migrated and non-migrated immune cells was determined by flow cytometry (Supplementary Figure S2). The presence of AEA alone decreased the migration rate of immune cells, while AEA in combination with Zymosan increased the percentage of migrated cells (Figures 3A, B). These data suggest that AEA can have a multifaceted role in immune cell migration. Similarly, the presence of AEA alone in the bottom chamber also decreased the migration rate of B cells (Figure 3C). AEA alone and in combination with Zymosan reduced the migration of NK cells (Figure 3D) and the cytotoxic NK cell subset (CD56dim CD16hi) (Figure 3E). However, there was no difference between groups in the migration rate of the CD56hi CD16dim NK cell subpopulation (Figure 3F). Importantly, the addition of AEA alone or together with Zymosan did not alter the migration rate of T cells *per se* (Figure 3G). However, there was a tendency for a reduced number of migrated CD4^+^ T cells (Figure 3H) towards AEA in combination with Zymosan, while AEA did not have impact on the migration rate of CD8^+^ T cells (Figure 3I) and CCR6+ CD4^+^ T cells (Figure 3J). When looking at myeloid cells, AEA did not affect the migration of myeloid cells *per se* (Figure 3K), as well as that of myeloid subsets such as classical monocytes (Figure 3L), intermediate monocytes (Figure 3M), mDCs (Figure 3N), and activated mDCs (Figure 3O). The migration of myeloid CD33low cells (Figure 3P), and CD195 (CCR5)+ myeloid CD33low cells (Figure 3Q), which may correspond to CD33^+^ NK cells, was decreased by AEA without the presence of Zymosan. Together, these data demonstrated that AEA directly affects migration of certain immune cell subsets, despite being applied at the site towards which the cells migrate.

### Anandamide stimulation alters the migratory capacity of human immune cells

**FIGURE 4:**
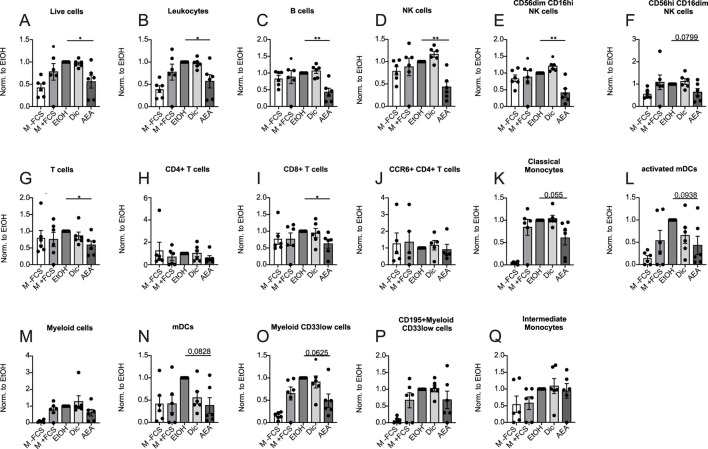
AEA treatment reduces immune cell migration. Human PBMCs were pre-treated with ethanol (EtOH), Diclofenac and EtOH (Dic), and AEA (10 µM) with Diclofenac (AEA). Medium without (-FCS) and with FCS (+FCS) were used as negative and positive controls. The numbers of migrated cells were measured by flow cytometry and represent three independent experiments. The numbers of live cells **(A)**, leukocytes **(B)**, B cells **(C)**, NK cells **(D)**, CD56dimCD16hi NK cells **(E)**, CD56hiCD16dim NK cells **(F)**, T cells **(G)**, CD4^+^ T cells **(H)**, CD8^+^ T cells **(I)**, CCR6+ CD4^+^ T cells **(J)**, classical monocytes **(K)**, activated mDCs **(L)**, myeloid cells **(M)**, mDCs **(N)**, myeloid CD33low cells **(O)**, CD195+myeloid CD33low cells **(P)**, and intermediate monocytes **(Q)** was calculated by the ratio of migrated/non-migrated cells and normalized to EtOH. Data are from three independent experiments, with 2 donors each. Each data point corresponds to a single donor (n = 6). Data are shown as mean ± SEM. *p < 0.05, **p < 0.01, ***p < 0.001, ****p < 0.0001; *p*-values were calculated using one-sample *t*-test.

Next, we tested whether or not pre-incubation of PBMCs with AEA would affect their ability to migrate towards FCS. Cells were pre-treated for 1 h with Diclofenac and for 2 h with AEA. Again, media with and without FCS were used as positive and negative controls respectively, and flow cytometry was employed to measure the migration rate of major immune cell populations. AEA dramatically reduced the migration rate of the cells studied including leukocytes (Figures 4A, B), B cells (Figure 4C), NK cells (Figure 4D) and cytotoxic NK cells (Figure 4E), while NK cell precursors treated with AEA demonstrated a tendency to reduced migration rate (Figure 4F). Importantly, pretreatment with AEA reduced migration of T cells (Figure 4G), where particularly the migration of CD8^+^ T cells (Figure 4I) treated with AEA was significantly reduced, while CD4^+^ T cells (Figure 4H) and CCR6+ CD4^+^ T cells (Figure 4J) did not respond to AEA treatment. Similarly, classical monocytes (Figure 4K), activated mDCs (Figure 4L), mDCs (Figure 4N), and myeloid CD33low cells (Figure 4O) treated with AEA showed a tendency towards lower migration rates. There was no difference in migrated cell number in myeloid cells (Figure 4M), CD195+ myeloid CD33low cells (Figure 4P), and intermediate monocytes (Figure 4Q) between groups. Collectively, these data indicate that AEA has a direct impact on immune cells and impairs their migration ability. Moreover, the data suggest that the increase in T cells in the animal model is not a consequence of an increased recruitment by AEA. Rather, AEA might block emigration of cells towards the lymphatics.

### AEA substantially affects proliferation, activation, and exhaustion status of CD4^+^ T cells

**FIGURE 5:**
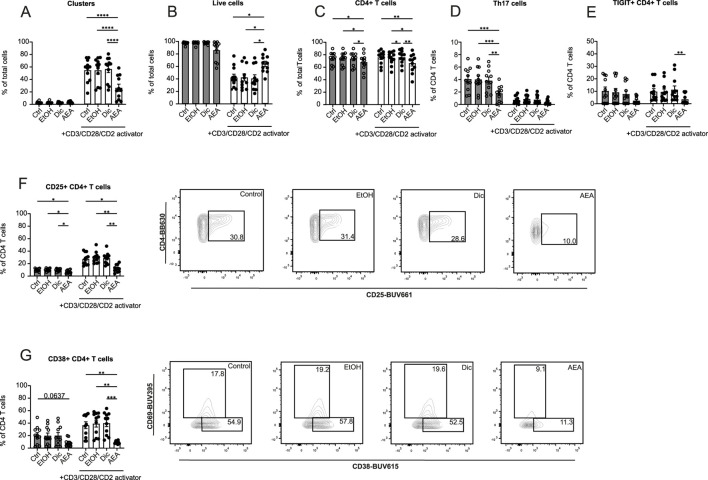
AEA dramatically alters the proliferation, activation and exhaustion profiles of CD4^+^ T cells. Human isolated T cells were pre-treated with ethanol (EtOH), Diclofenac and EtOH (Dic), and AEA (10 µM) with Diclofenac (AEA), and were activated or remained inactivated for up to 6 days. Cells were measured by flow cytometry. Percentage of cell clusters **(A)**, live cells **(B)**, CD4^+^ T cells **(C)**, Th17 cells **(D)**, TIGIT+CD4^+^ T cells **(E)**, CD25^+^ CD4^+^ T cells **(F)**, and CD38^+^CD4^+^ T cells **(G)** are shown. Data are from three independent experiments, with at least 3 donors each. Each data point corresponds to a single donor (n = 11). Data are shown as mean ± SEM. *p < 0.05, **p < 0.01, ***p < 0.001, ****p < 0.0001; *p*-values were calculated using two-way ANOVA with Tukey correction.

Following the findings that AEA altered T cell migration in mouse and human systems, the question whether or not AEA may affect the T cell phenotype at sites of inflammation was studied. Bead-isolated human T cells were treated with AEA or control and were afterward activated with CD3/CD28/CD2 activator cocktail for 5 days. Using flow cytometry, the expression levels of chemokine receptors (CCR6, CCR2, CXCR3, CXCR4), activation markers (CD25, CD38, CD69), and exhaustion markers (TIGIT, LAG3, PD1) were measured (Supplementary Figures S3, S4). Similar to the macrophage experiments, AEA was tested at concentrations 10 nM and 10 µM. Anandamide at lower concentrations did not have any impact on T cell activation, proliferation, and maturation status. However, a marked reduction in T cell cluster formation was observed (Figure 5A) when the cells were treated with 10 µM AEA. In contrast, the percentage of live T cells increased in the presence of AEA (Figure 5B). Both parameters indicate reduced T cell activation. There was no difference in clusters and live cell numbers between groups in non-activated T cells. Addition of AEA decreased percentage of CD4^+^ T cell among non and activated cells (Figure 5C). Surprisingly, a significant reduction in non-activated Th17 cells treated was observed in the AEA treated group (Figure 5D). Additionally, AEA decreased the percentage of activated TIGIT+CD4^+^ T cells (Figure 5E). AEA also reduced the expression of CD38 and CD25, both in activated and non-activated CD4^+^ T cells (Figures 5F, G). No alterations were identified in CCR2+ CD4^+^ T cells, LAG3+ CD4^+^ T cells, Th1 cells, and Th2 cells (Supplementary Figure S5). These data demonstrated that AEA decreases the activation and exhaustion status of CD4^+^ T cells and reduces the number of Th17 cells.

### AEA alters the proliferation, activation and exhaustion status of CD8^+^ T cells

**FIGURE 6:**
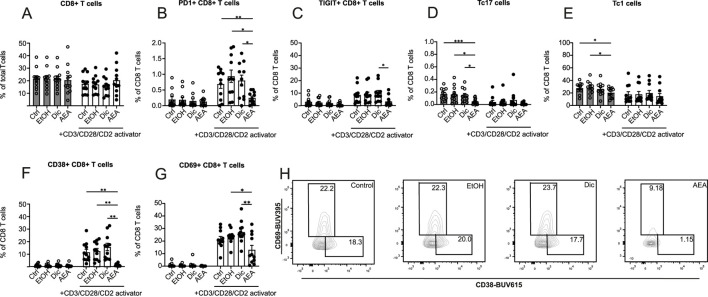
AEA has an impact on the proliferation, activation and exhaustion profiles of CD8 T cells. Human isolated T cells were pre-treated with ethanol (EtOH), Diclofenac and EtOH (Dic), and AEA (10 µM) with Diclofenac (AEA), and were activated or remained inactivated for up to 6 days. Cells were measured by flow cytometry. Percentage of CD8^+^ T cells **(A)**, PD1+ CD8^+^ T cells **(B)**, TIGIT+ CD8^+^ T cells **(C)**, Tc17 cells **(D)**, Tc1 cells **(E)**, CD38^+^ CD8^+^ T cells **(F)**, and CD69^+^CD8^+^ T cells **(G)** are shown. **(H)** Representative FACS plot of CD69^+^CD8^+^ T and CD38^+^CD8^+^ T cells. Data are from three independent experiments, with at least 3 donors each. Each data point corresponds to a single donor (n = 11). Data are shown as mean ± SEM. *p < 0.05, **p < 0.01, ***p < 0.001, ****p < 0.0001; *p*-values were calculated using two-way ANOVA with Tukey correction.

The impact of AEA on CD8^+^ T cells was measured alongside CD4^+^ T cells. By contrast, AEA was found not to alter the percentage of CD8^+^ T cells (Figure 6A). When looking closer at CD8^+^ T cell activation and exhaustion status, PD1+ CD8^+^ T cells (Figure 6B), TIGIT+ CD8^+^ T cells (Figure 6C), CD38^+^ CD8^+^ T cells (Figures 6F, H), and CD69^+^ CD8^+^ T cells (Figures 6G, H) populations were significantly decreased following AEA and activator addition. Similarly, to CD4^+^ T cells, a reduced Tc17 cells percentage in the AEA group in non-activated cells was observed (Figure 6D). Moreover, AEA presence decreased the Tc1 percentage in the non-activated group (Figure 6E). There was no observable effect on CCR2+ CD8^+^ T cells, LAG3+ CD8^+^ T cells, and Tc2 cells (Supplementary Figure S5). Taken together, these data demonstrated that AEA reduces the number of activated and exhausted CD8^+^ T cells and limits CD8^+^ T cell subsets such as Tc1 and Tc17 cells.

### Anandamide reduces the expression of IL-10, IL-17a, and IL-13 in activated human T cells

**FIGURE 7:**
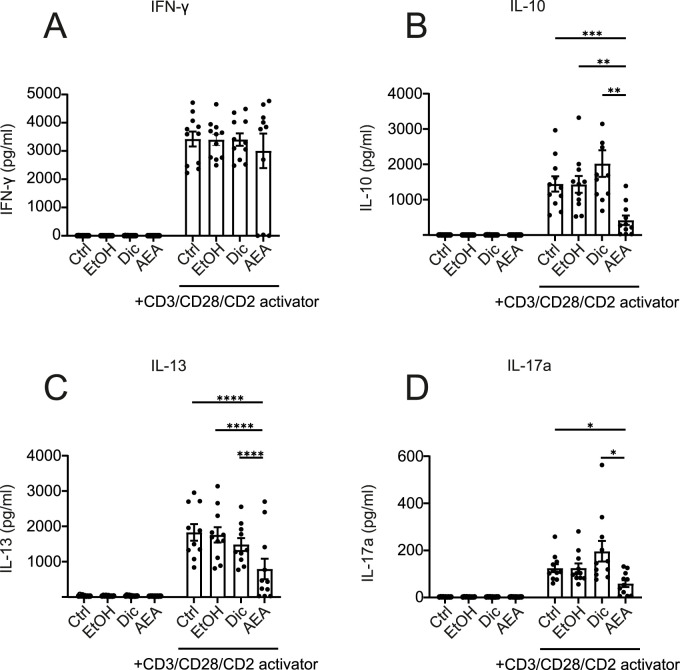
AEA reduces the levels of IL-10, IL-13, and IL-17A in activated human T cells. Human isolated T cells were pre-treated with ethanol (EtOH), Diclofenac and EtOH (Dic), and AEA (10 µM) with Diclofenac (AEA), and were activated or remained inactivated for up to 6 days. Supernatant was harvested and cytokines were measured using Cytometric Bead Array by flow cytometry. The concentrations of IFN-γ **(A)** IL-10 **(B)**, IL-13 **(C)**, and IL-17A **(D)** are shown. Data are from three independent experiments, with at least 3 donors each. Each data point corresponds to a single donor (n = 11). Data are shown as mean ± SEM. *p < 0.05, **p < 0.01, ***p < 0.001, ****p < 0.0001; *p*-values were calculated using two-way ANOVA with Tukey correction.

Based on Anandamide altering T cell activation surface markers, we further investigated if it would affect T cell cytokine production. To test this, we collected supernatants after T cells were treated with AEA and activated for 6 days. Despite reduced activation observed by flow cytometry, there was no significant difference in interferon-γ (IFN-γ) secretion (Figure 7A). However, we found a dramatic reduction in IL-10, IL-13, and IL-17A cytokine production following AEA and activator addition (Figures 7B–D). These data confirmed that AEA has an impact on T cell activation. Particularly, AEA decreases the production of Th2 and Th17 cytokines, but does not alter the Th1 cytokine IFN-γ. Previous studies showed that AEA at already low concentrations engages CB2 receptors in immune cells altering cell activation and differentiation (Cencioni et al., 2010). Therefore, we measured IL-10, IL-17A, IL-13, and IFN-γ cytokine production in activated T cells treated with AEA at concentrations of 10 nM, 100 nM, and 1 µM (data not shown), which would be sufficient for CB receptor engagement. However, we did not observe any effect in cytokine production when T cell were treated with lower concentrations of AEA. These data suggest a shift in T cell activation profiles rather than a global suppression of T cell activation by AEA. While AEA exhibits its central effects primarily through Gi-coupled cannabinoid receptors CB1 and CB2 receptors which are activated already at nanomolar concentrations (Munro et al., 1993), the lack of an anti-inflammatory effect at such a low concentration suggests alternative receptors with lower sensitivity like intracellular receptors. These require efficient cellular uptake and thus particularly high extracellular concentrations (Teichmann et al., 2024).

### Anandamide increases T-cell through NR4A1

**FIGURE 8:**
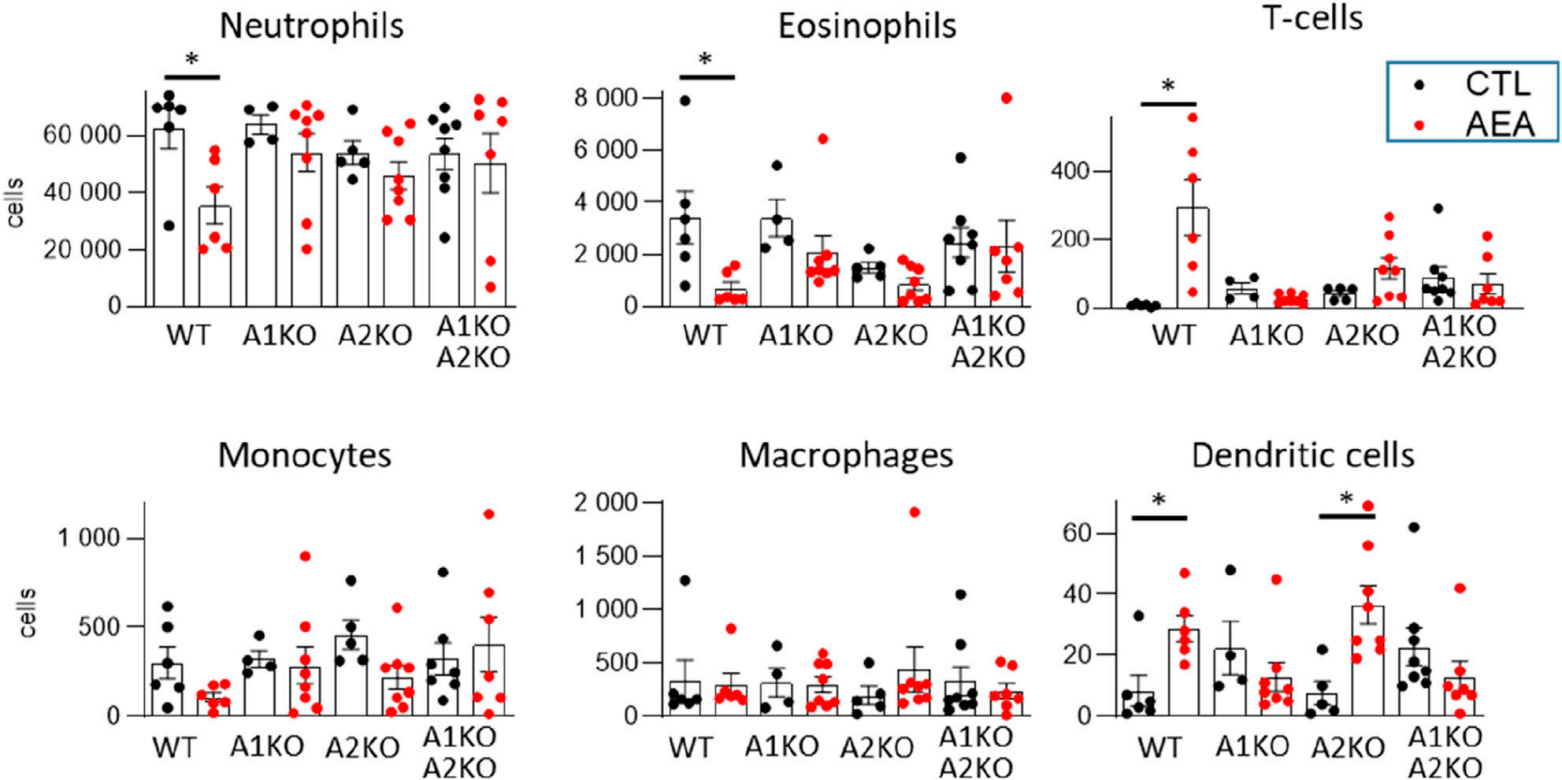
AEA increases T-cell number through NR4A receptors in the AirPouch Zymosan model. Analysis of cellular infiltrate in murine AirPouch exudates after control (CTL) or AEA nanoformulation (AEA) pre-treatment (1 h) followed by zymosan stimulation (6 h) in mice inheriting a knockout for NR4A1^−/−^ (A1KO), NR4A2^−/−^ (A2KO) or double knockout for NR4A1/2^−/−^, compared to control mice (also shown in Figure 1). Mice without knockout on the floxed/cre background served as control (WT). Each data point represents an animal showing the total number of cells recovered by FACS analysis. 1-way ANOVA and Mann-Whitney test. n = 4–8. *p < 0.05.

Based on our previous study suggesting that NR4A1 and NR4A2 may contribute to the signaling of AEA, and the importance of the NR4A family in T cell biology (Odagiu et al., 2020), the effect of genetic deletion of each receptor or both in combination was determined in the AirPouch model. Deletion of any of the two receptors blocked the effect of AEA. NR4A1 and NR4A2 deficient, as well as double knockout mice, did not exhibit a significant decrease in neutrophil or eosinophil accumulation. Although not statistically significant, it appeared that knockout of each receptor partially attenuated the response of AEA with the double knockout mouse exhibiting the strongest, indicating additive reduction (Figure 8).

Double knockout of NR4A1 and NR4A2 also prevented the AEA-induced increase in T cell and dendritic cells. A similar effect was observed in NR4A1 KO mice. In NR4A2 KO mice, AEA still induced an increase in T cells and dendritic cells, which for T cells did, however, not reach the significance level with the group size present (Figure 8). Collectively, these data suggest that AEA elicits an inhibitor effect on granulocyte accumulation in the AirPouch model by NR4A1 and NRA2, whereas it increases T cell and dendritic cell accumulation through NR4A.

## Discussion

Anandamide was the first endocannabinoid described to activate CB1 and CB2 receptors. AEA is pivotal in regulating inflammation as indicated by recent studies showing that AEA administration lessens inflammation and improves survival scores in various inflammatory murine models (Berg et al., 2023; Sultan et al., 2021). Nevertheless, its impact on immune cell migration and activation in murine and human systems remain controversial. We found in this study that AEA application in the Zymosan AirPouch mouse model decreases the infiltration of neutrophils, eosinophils, and monocytes to the site of inflammation, and elevates the number of T cells in the Zymosan-enriched area. To explore this issue, we employed a human *in vitro* system to gain a better understanding of the immune cell migration process.

It was shown that cannabinoids can have anti-inflammatory effects on primary human macrophages (Gojani et al., 2023) and activated microglial cells (Rodrigues et al., 2024). Moreover, Leuti *et al.* showed that AEA promotes pro-resolving mechanisms in human macrophages by binding to CB2 and GPR18 receptors (Leuti et al., 2024). In our study AEA impaired chemokine production in Zymosan-activated macrophages, particularly of CCL2, CCL4, CCL17, and CCL20. Together, these chemokines are responsible for myeloid and lymphocyte cell infiltration into the sites of inflammation, suggesting that AEA can reduce severity in the acute phase of inflammatory diseases. In particular, CCL2 plays a crucial role in the attraction of various immune cell subsets (monocytes, memory T cells, and DCs) to the inflamed region by engaging the CCR2 receptor (Gschwandtner et al., 2019). CCL4 is known to be a pro-inflammatory chemokine and one of the main chemoattractants for CD8^+^ T cells (Castellino et al., 2006). Similarly, Th2 cells accumulate in response to CCL17 via the CCR4 receptor, contributing to allergic pathological conditions (McIlroy et al., 2006). The reduced level of CCL20 upon AEA treatment is of particular interest, given that CCL20 is the major chemokine for Th17 migration to the inflammatory milieu by engaging its receptor CCR6 on the Th17 cell surface, and the CCR6-CCL20 axis is involved in the pathogenesis of various chronic inflammatory and autoimmune diseases (Meitei et al., 2021). It was previously shown that AEA is responsible for keratinocyte-dependent inhibition of Th1 and Th17 polarization (Chiurchiù et al., 2016). An inhibitory role of AEA on chemokine production was described previously. AEA reduced the production of CXCL8 in monocyte-derived Langerhans cells induced by TLR7/8 (Pénzes et al., 2024), and CCL2 levels in vascular smooth muscle cells (Pflüger-Müller et al., 2020) and keratinocytes (McCormick et al., 2023). The current study adds to these data by showing that AEA alters the expression of CCL2, CCL4, CCL17, and CCL20 in human macrophages that are activated via TLR2 and TLR6 signaling pathways.

AEA alone or in combination with Zymosan was found to inhibit the migration of B cells and NK cell subsets. Based on the observations that B cells and NK cells exposed to AEA exhibit reduced migration, we can conclude that AEA directly influences these lymphocyte populations by impairing their motility. Previous studies have shown that lower concentrations of AEA decrease NK cell cytotoxic activity *in vitro* (Lissoni et al., 2008), but did not affect NK cell line motility (Kishimoto et al., 2005). Research on the effect of cannabinoids and ECS on B cells is limited. A recent study showed that cannabinoids have a dramatic effect on the cytokine profile of B cells, and reduce the number of IgM+/IgG+ cells (Lampron et al., 2023). Also, Tetrahydrocannabinol (THC) and AEA were shown to decrease antibody formation in plaque-forming cell assays (Eisenstein et al., 2007). Intriguingly, the endocannabinoid 2-AG induced B cell migration among murine splenocytes *in vitro* (Alberich Jordà et al., 2002). The present data add to these findings, by showing that AEA directly influences NK and B cell migration, which can be important to further investigate the impact of AEA in the fields of autoimmune disease and cancer.

Previous studies have investigated an impact of AEA on T cell activation and cytokine production *in vitro* both in human and murine settings (Cencioni et al., 2010; Ribeiro et al., 2010; Sultan et al., 2021). Similar to prior findings (Cencioni et al., 2010), we confirm that AEA decreased the production of IL-17A in activated human T cells. Nevertheless, we did not observe reduced levels of IFN-γ upon AEA treatment. By contrast, we determined that AEA does not have any impact on IFN-y production, while it dramatically decreased IL-10 and IL-13 in T cell-derived supernatants. This indicates that AEA possibly alters Th2, Th17, and Treg differentiation, while Th1 differentiation remains unchanged. Differences to previous studies can be explained due to the different functional read-outs. The present study focused on T cell differentiation (culture of activated T cells for 6 days) rather than acute cytokine production in activated T cells triggered by protein kinase C activation as determined before (Cencioni et al., 2010). Previously, it was shown that AEA is oxidized by COX-2 (Yu et al., 1997). Here, the COX-2-dependent breakdown of AEA was blocked, enabling longer culture protocols.

Surprisingly, given the selected changes in cytokine production, T cell activation as determined by cell cluster formation and increased numbers of live activated T cells death (Green et al., 2003) was broadly reduced upon AEA administration. Additionally, the expression of activation receptors on the T cell surface was dramatically decreased in the presence of AEA. Decreased CD25, but not CD69 expression on CD4^+^ T cells treated with AEA was described before (Sultan et al., 2021). The findings described in here add that AEA also decreased CD38 expression on CD4^+^ T cells, as well as CD38 and CD69 expression on CD8^+^ T cells.

Recently, there has been a growing interest in the role of endocannabinoids and cannabinoids in tumor progression and their impact on the efficacy of immunotherapeutic drugs (Sarsembayeva and Schicho, 2023). AEA was previously shown to reduce the effect of PD-1 antibodies in tumor mouse models (Xiong et al., 2022). This work shows that AEA decreases the expression of PD-1 on the surface of activated CD8^+^ T cells, and TIGIT on the surface of activated CD4^+^ T and CD8^+^ T cells. TIGIT is known to inhibit anti-tumor immunity (Tang et al., 2023). Thus, these data may shed light on the involvement of endocannabinoids and possible therapeutic approaches for cancer treatment.

Beside cancer, the data present here provide insights into a potential role of AEA in acute and chronic inflammatory settings. Given the contrast between murine data and results derived from migration assays and T cell activation, the increased number of T cells in the AirPouch model can be explained by their reduced activation status and motility, which causes the T cells to be retained at the site of inflammation, instead of remigrating towards lymphatics. This is consistent with data on DCs in the AirPouch model since these cells follow comparable chemokine gradients towards the lymphatics compared to T cells (Johnson and Jackson, 2014). Granulocytes and myeloid cells are recruited to an inflamed region before lymphocyte recruitment, since these cells are responsible for the first line defense. Thus, AEA would act on recruitment of these myeloid cells from the circulation at the early time point employed in the AirPouch model in this study, but not on lymphocyte recruitment, rather acting on tissue-resident lymphocytes (Ziessman et al., 2006). T cells accumulating locally upon AEA treatment likely experience altered activation and, subsequently exhaustion due to AEA. Previously, it was shown that proliferation of T cells toward Th17 cells is inhibited by AEA (Cencioni et al., 2010). By contrast, polarization toward the Th1 phenotype was facilitated indirectly via AEA (Pénzes et al., 2024). These findings of a preserved Th1, but blocked Th17 differentiation are supported by the data presented herein. Thus, T cells accumulating locally under the influence of AEA appear to remain competent in fighting infection, while chronic inflammatory responses are prevented. These features position AEA as a player promoting resolution of infection and inflammation, by ensuring the removal of the inflammatory trigger but blocking chronification of inflammation. This is supported by the findings that AEA blocked the expression of CCL20, a major Th17 chemokine. Overall, AEA emerges as a negative regulator of chronic inflammatory T cell activation and recruitment.

The following experiments with NR4A1 and NR4A2 KO mice in the AirPouch model demonstrated that AEA engaging NR4A1 and NR4A2 receptors is responsible for decreased eosinophils and via NR4A1 increased T-cell and DC accumulation. Previous data showed that NR4A1 upregulation caused T cell dysfunction (Liu et al., 2019). Whether this is connected to the action of AEA remains to be determined in future studies. Taken together, the data presented here demonstrate that AEA has a strong inhibitory effect on T cell activation, while increasing the number of T cells at the site of inflammation via the NR4A1 receptor. A deeper understanding of the mechanisms underlying the interaction between AEA and nuclear receptors in T cells may shed light on therapeutic uses for AEA in chronic inflammatory disease settings.

## Data Availability

The original contributions presented in the study are included in the article/Supplementary Material, further inquiries can be directed to the corresponding authors.
