# Supplementary material for: The endocannabinoid anandamide prevents TH17 programming of activated T lymphocytes while preserving TH1 responses
Source: Front Pharmacol. 2024 Dec 20;15:1528759. doi: 10.3389/fphar.2024.1528759 (PMC11695368; doi:10.3389/fphar.2024.1528759)
Supplement: Supplementary file 1 [file DataSheet1.pdf]

Supplementary Figures and Figure Legends

Figure S1

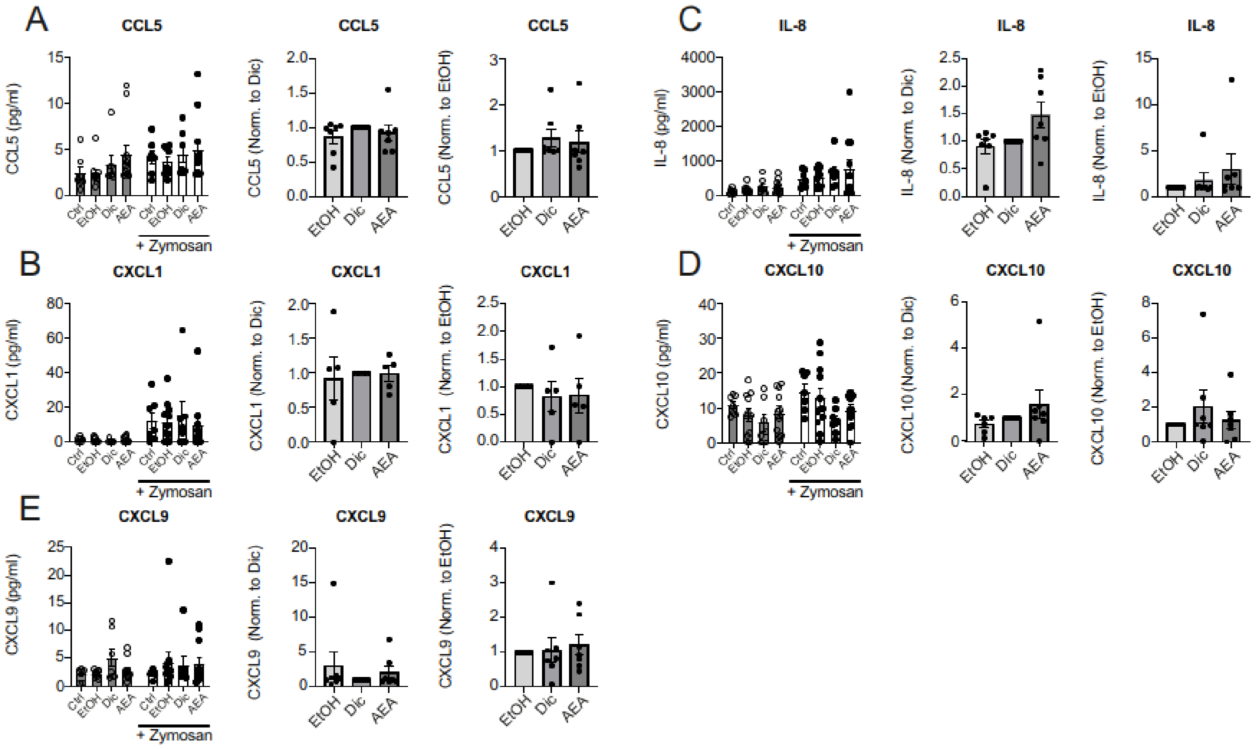

Figure S1. Anandamide reduces chemokine production in Zymosan-activated Macrophages. Human primary macrophages were treated with Ethanol (EtOH), Diclofenac and EtOH (Dic), or Diclofenac and AEA, and were subsequently stimulated with Zymosan for 6 h. The concentrations of CCL5 (A), CXCL1 (B), IL-8 (C), CXCL10 (D), CXCL9 (E) were determined by LegendPlex assay. Data are from three independent experiments. Besides raw data, normalized data of Zymosan-activated cells is shown. Each data point corresponds to a single donor. Data are shown as mean ± SEM. *p<0.05, **p<0.01, ***p<0.001, ****p<0.0001; p-values were calculated using two-way ANOVA or one-sample t-test.

Figure S2

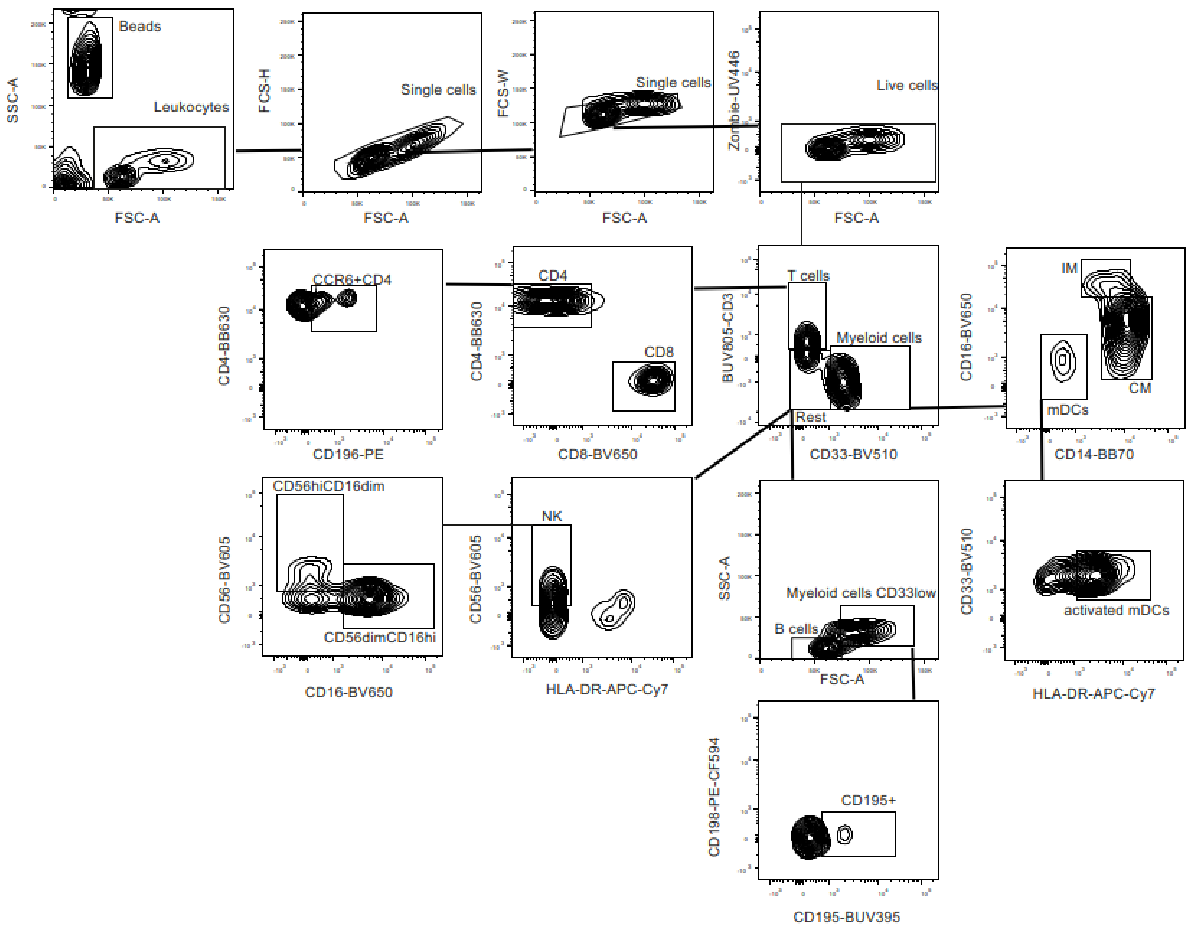

Figure S2. Representative FACS plots of Migration assay gating strategy.

Figure S3

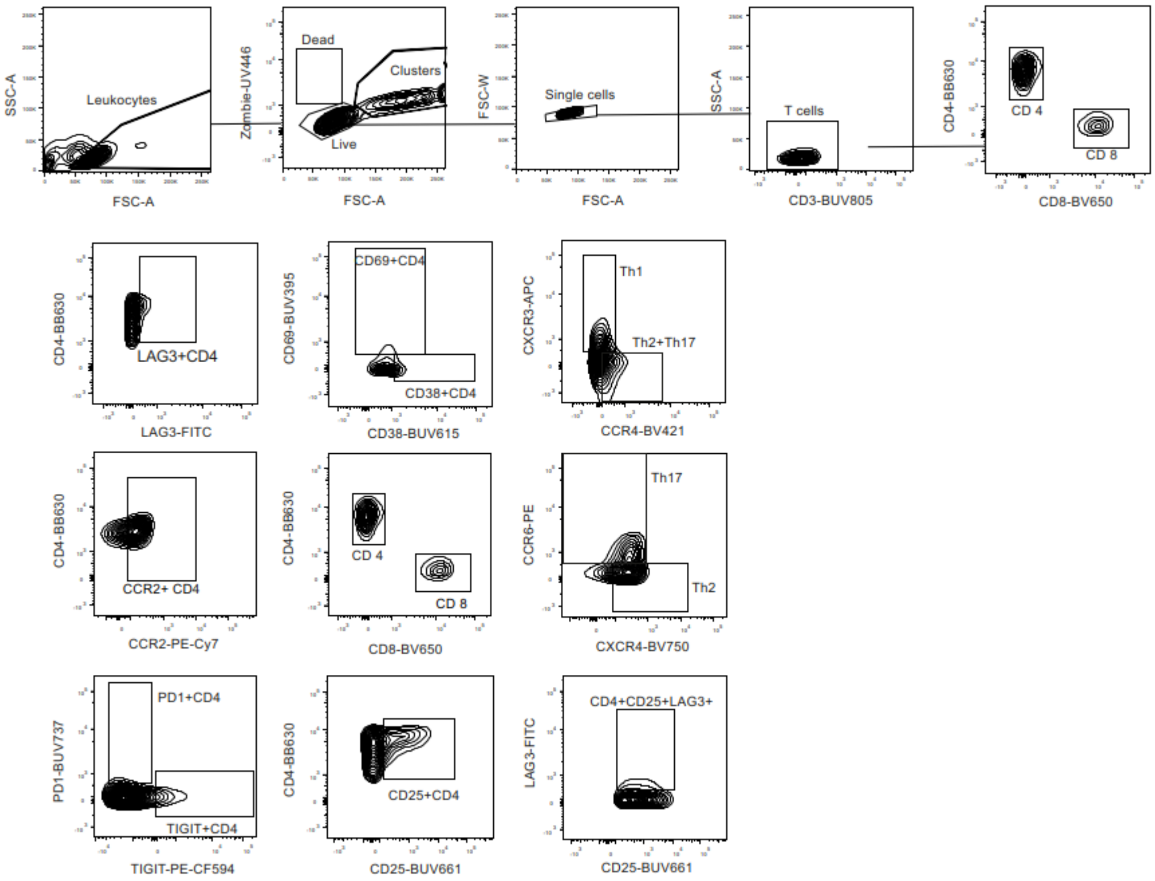

Figure S3. Representative FACS plots of Anandamide treated CD4 T cells assay gating strategy.

Figure S4

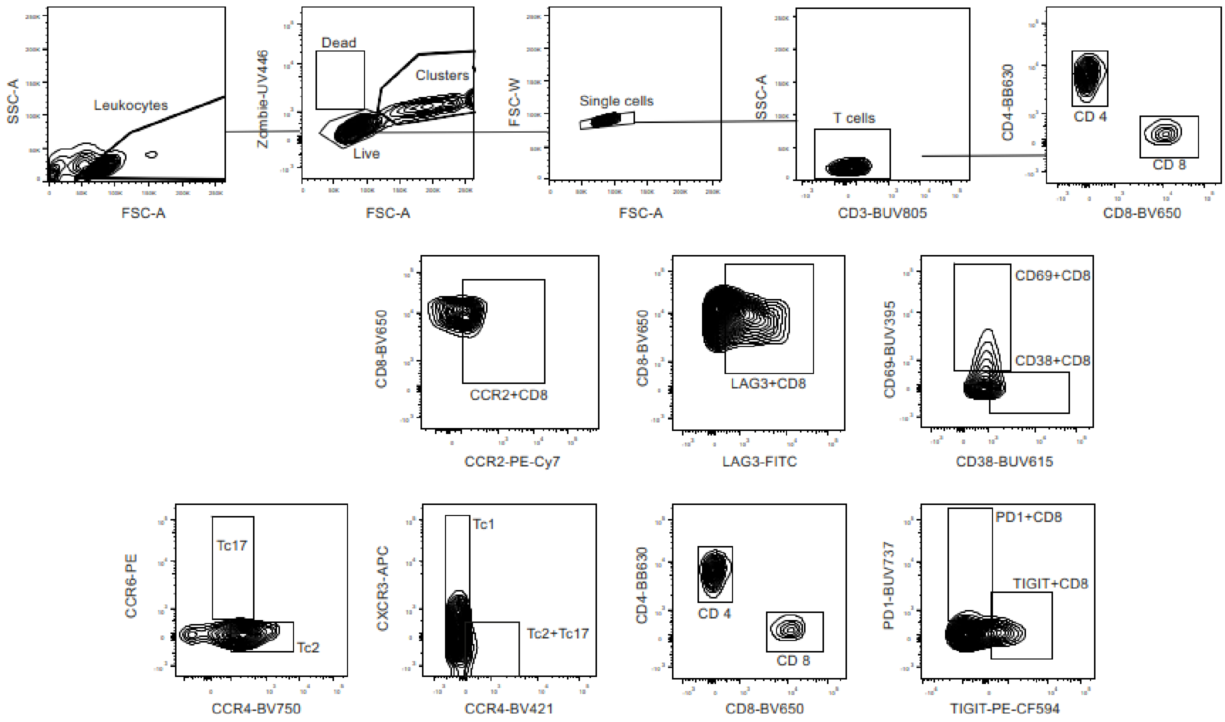

Figure S4. Representative FACS plots of Anandamide treated CD8 T cells assay gating strategy.

Figure S5

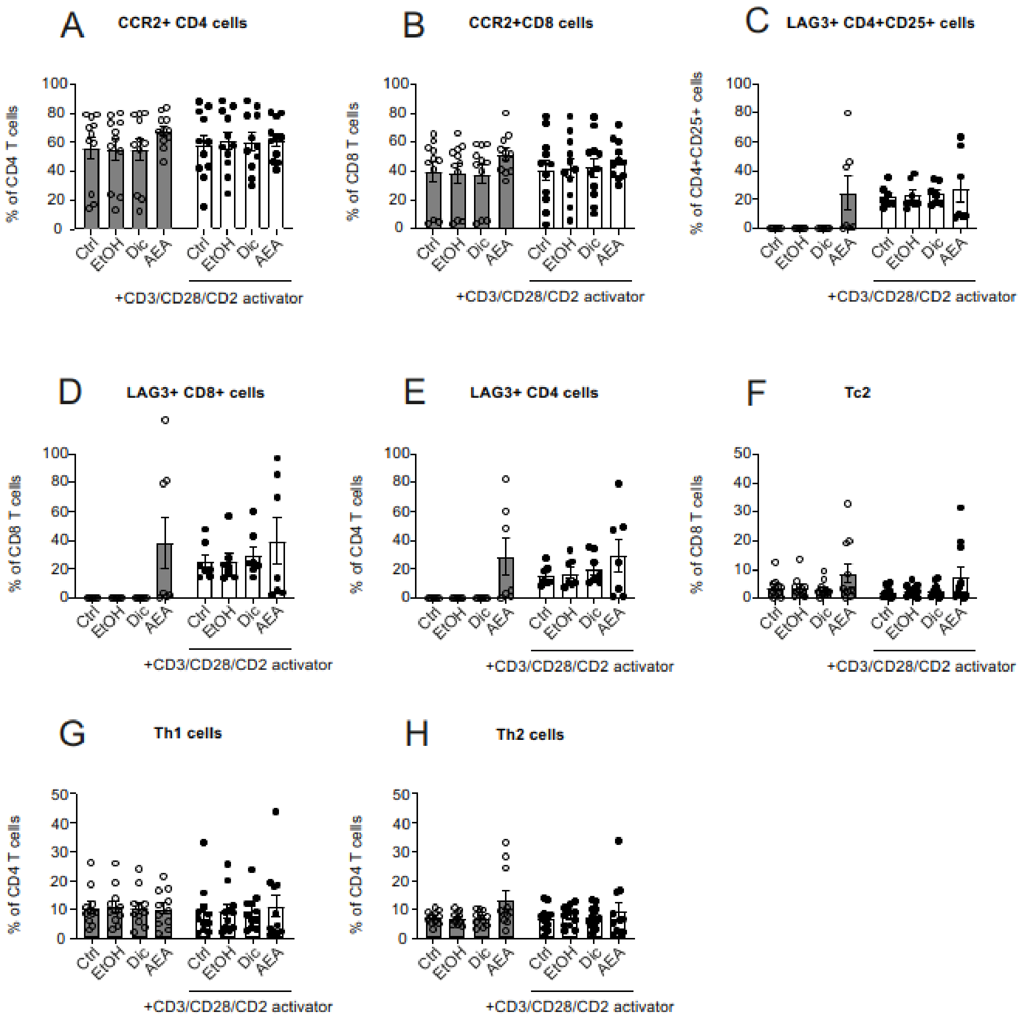

Figure S5. Anandamide alters the proliferation, activation, and exhaustion profiles of T cells. Human isolated T cells were pre-treated with Ethanol (EtOH), Diclofenac and EtOH (Dic), and AEA with Diclofenac (AEA), and were activated or remained inactivated for up to 6 days. Cells were measured by flow cytometry. Percentage of CCR2+CD4 (A), CCR2+CD8 (B), LAG3+CD4+CD25+(C), LAG3+CD8+ (D), LAG3+CD4 cells (E), Tc2 (F), Th1 (G), Th2 (H) cells are shown. Data represent three independent experiments, and each data point corresponds to a single donor. Data are shown as mean ± SEM. *p<0.05, **p<0.01, ***p<0.001, ****p<0.0001; p-values were calculated using two-way ANOVA with Tukey correction.
